# Fibroblast growth factor receptor facilitates recurrence of minimal residual disease following trastuzumab emtansine therapy

**DOI:** 10.1038/s41523-020-00213-5

**Published:** 2021-01-21

**Authors:** Saeed S. Akhand, Hao Chen, Stephen Connor Purdy, Zian Liu, Joshua C. Anderson, Christopher D. Willey, Michael K. Wendt

**Affiliations:** 1 Purdue University Center for Cancer Research, Department of Medicinal Chemistry and Molecular Pharmacology, Purdue University, West Lafayette, IN 47907 USA; 2 Department of Radiation Oncology, University of Alabama at Birmingham, Birmingham, AL 35244 USA

**Keywords:** Cancer therapeutic resistance, Cancer models, Breast cancer, Receptor pharmacology, Targeted therapies

## Abstract

Trastuzumab-emtansine (T-DM1) is an antibody-drug conjugate (ADC) that efficiently delivers a highly potent microtubule inhibitor to HER2 overexpressing cells. Herein, we utilize HER2 transformed human mammary epithelial cells (HME2) to demonstrate in vitro and in vivo response and recurrence upon T-DM1 treatment. Continuous in vitro dosing of HME2 cells with T-DM1 failed to produce a spontaneously resistant cell line. However, induction of epithelial–mesenchymal transition (EMT) via pretreatment with TGF-β1 was capable of promoting emergence of T-DM1-resistant (TDM1R) cells. Flow cytometric analyses indicated that induction of EMT decreased trastuzumab binding, prior to overt loss of HER2 expression in TDM1R cells. Kinome analyses of TDM1R cells indicated increased phosphorylation of ErbB1, ErbB4, and FGFR1. TDM1R cells failed to respond to the ErbB kinase inhibitors lapatinib and afatinib, but they acquired sensitivity to FIIN4, a covalent FGFR kinase inhibitor. In vivo, minimal residual disease (MRD) remained detectable via bioluminescent imaging following T-DM1-induced tumor regression. Upon cessation of the ADC, relapse occurred and secondary tumors were resistant to additional rounds of T-DM1. These recurrent tumors could be inhibited by FIIN4. Moreover, ectopic overexpression of FGFR1 was sufficient to enhance tumor growth, diminish trastuzumab binding, and promote recurrence following T-DM1-induced MRD. Finally, patient-derived xenografts from a HER2^+^ breast cancer patient who had progressed on trastuzumab failed to respond to T-DM1, but tumor growth was significantly inhibited by FIIN4. Overall, our studies strongly support therapeutic combination of TDM1 with FGFR-targeted agents in HER2^+^ breast cancer.

## Introduction

Human epidermal growth factor receptor 2 (HER2) is a member of the ErbB family of receptor tyrosine kinases. HER2-amplified breast cancers respond to treatment with the HER2-targeted monoclonal antibodies pertuzumab and trastuzumab at a high rate, but acquired resistance to these therapies remains a major clinical problem for patients with this breast cancer subtype. Trastuzumab-emtansine (T-DM1) is an antibody-drug conjugate (ADC) that provides a mechanism to deliver a potent microtubule-targeting cytotoxin to HER2 overexpressing cells. Initial enthusiasm for T-DM1 based on dramatic preclinical results has been somewhat tempered by the inability of T-DM1 to improve patient outcomes as compared to trastuzumab and a taxane in the first line setting^1,2^. While several more recent trials have found T-DM1 to be effect in the later line settings, disease recurrence and progression is still a major clinical issue. These data suggest that there are uncharacterized drivers of resistance at play^3^.

Epithelial-mesenchymal transition (EMT) is a normal physiological process whereby polarized epithelial cells transition into motile, apolar fibroblastoid-like cells to facilitate several developmental events and to promote wound repair in response to damaged tissues^4^. In contrast, initiation of pathological EMT engenders the acquisition of invasive, metastatic, and drug-resistant phenotypes to developing and progressing carcinomas^5–7^. Physiologic and pathologic EMT can be induced by cytokines such as TGF-β and HGF^8^. More recent findings demonstrate that EMT can be initiated by treatment with kinase inhibitors and that this transition to a mesenchymal state facilitates tumor cell persistence in the sustained presence of these molecular-targeted compounds^9^. In contrast to kinase inhibition, very little is known about the mechanisms by which EMT may facilitate resistance to antibody and ADC therapies.

Induction of EMT increases the expression of fibroblast growth factor receptor 1 (FGFR1)^10,11^. FGFR1 can also undergo gene amplification and translocation, and elevated expression of FGFR1 is associated with decreased clinical outcomes of breast cancer patients^12–14^. Work from our lab and others suggest that upregulation of FGFRs and FGF ligands can serve as resistance mechanisms for tumor cells that were originally sensitive to ErbB and endocrine-targeted therapies^14–18^. In addition to enhanced expression of the receptor, our recent studies demonstrate that the processes involved in EMT work en masse to support FGFR signaling through diminution of E-cadherin and enhanced interaction with integrins^19^. Several different Type I, ATP-competitive kinase inhibitors against FGFR have been developed, and we and others have demonstrated their in vivo efficacy in delaying the growth of metastatic breast cancers^10,20,21^. Based on the potential of FGFR as a clinical target for cancer therapy, we recently developed FIIN4, a highly specific and extremely potent covalent kinase inhibitor of FGFR, capable of in vivo tumor inhibition upon oral administration in rodent models^18,19^.

In the current study, we address the hypothesis that FGFR can act as a driver of resistance to T-DM1. The use of in vivo and in vitro models demonstrate that unlike ErbB-targeted kinase inhibitors, EMT cannot overtly elicit resistance to T-DM1. Instead, induction of EMT and upregulation of FGFR1 induce a cell population with reduced trastuzumab binding. This minimal residual disease (MRD) is able to persist in the presence of T-DM1 and eventually reemerge as recurrent tumors where FGFR acts as a major driver of growth. Overall, our studies strongly suggest that combined therapeutics targeting HER2 and FGFR will delay tumor recurrence and prolong response times of patients with HER2^+^ breast cancer.

## Results

### T-DM1 resistance following induction of MRD

**Fig. 1:**
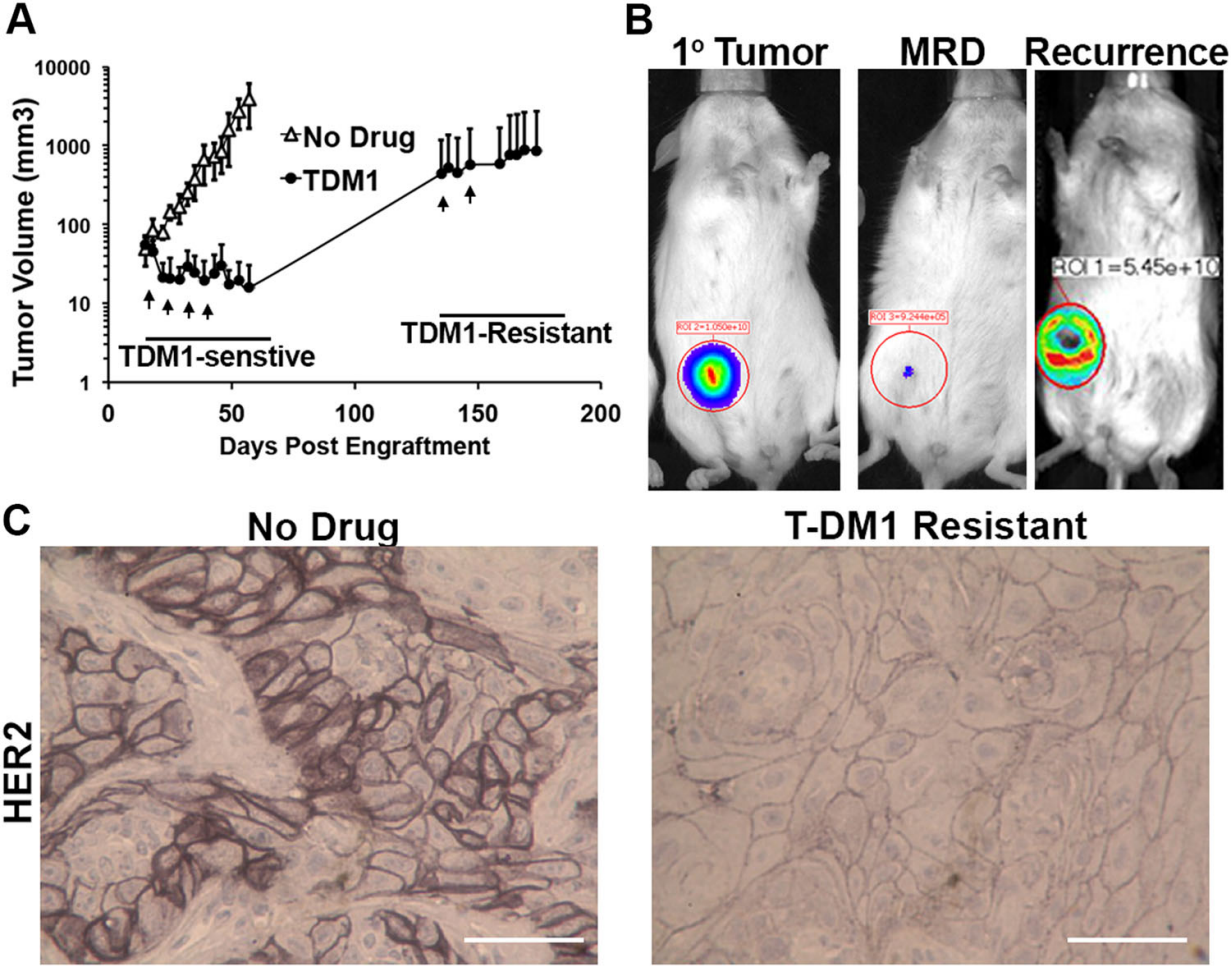
T-DM1 resistance following induction of minimal residual disease. **A** Mice were inoculated with HME2 cells (2 × 10^6^ cells/mouse) via the mammary fat pad and tumors were allowed to form for a period of 14 days. At this point, mice were split into two cohorts (5 mice/group) and left untreated (no drug) or were treated with T-DM1 (9 mg/kg) at the indicated time points (arrows). Following complete regression of palpable tumors, T-DM1 treatment was stopped. Recurrent tumors (3 mice) were again treated with T-DM1 (arrows). **B** Representative bioluminescent images of tumor-bearing mice before T-DM1 treatment (1° tumor), following T-DM1 treatment (MRD), and upon tumor recurrence. **C** Immunohistochemistry for HER2 expression in control and recurrent, T-DM1-resistant HME2 tumors.

Human mammary epithelial (HMLE) cells can be transformed by overexpression of wild-type HER2 (refs ^18,22^). Additionally, HER2 overexpression allows these cells to be cultured outside of the defined growth factor rich mammary gland media required for HMLE culture. In addition to antibiotic selection, this change from a defined media to FBS-containing media prevents the growth of non-HER2 expressing cells, driving a uniform HER2^+^ culture that is highly sensitive to inhibition of HER2 (ref. ^18^). Engraftment of these HER2-transformed HMLE cells (HME2) onto the mammary fat pad results in robust formation of highly differentiated, nonmetastatic, secretory tumors that demonstrate robust HER2^+^ expression consistent with that of HER2^+^ patient tumors (Supplementary Fig. 1; ref. ^23^). Here, we engrafted the HME2 cells onto the mammary fat pad and upon formation of orthotopic tumors mice received four intravenous injections of T-DM1 administered once a week for 4 weeks (Fig. 1A,B). This treatment protocol led to robust regression of these tumors to a point which they were no longer palpable and therefore immeasurable by digital calipers (Fig. 1A). However, these HME2 cells were constructed to stably express firefly luciferase and MRD was still detectable via bioluminescent imaging (Fig. 1B). Cessation of T-DM1 treatment led to recurrence of mammary fat pad tumors in three of five mice over approximately a 150-day period (Fig. 1A). Importantly, these recurrent tumors were nonresponsive to additional rounds of T-DM1 (Fig. 1A). Histological assessment of the recurrent tumors clearly demonstrated reduced levels of HER2 as compared to the untreated HME2 tumors (Fig. 1C). Overall these data demonstrate that even cells specifically transformed by HER2 overexpression are capable of establishing T-DM1 persistent MRD and undergoing drug-resistant recurrence.

### In vitro establishment of T-DM1-resistant cells requires prior induction of EMT

**Fig. 2:**
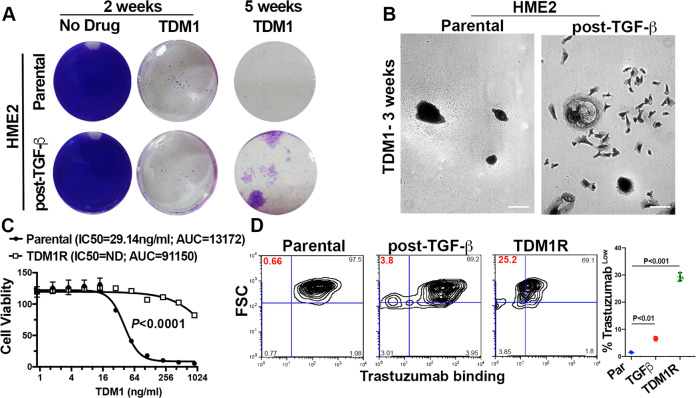
In vitro establishment of T-DM1-resistant cells requires prior induction of epithelial–mesenchymal transition. **A** HME2 cells were left untreated (parental) or were stimulated with TGF-β1 and allowed to recover (post-TGF-β) as described in the “Materials and methods”. These two cell populations were subsequently treated with T-DM1 (250 ng/ml) every 3 days for a period of 5 weeks. Representative wells were stained with crystal violet at the indicated time points to visualize viable cells. **B** Brightfield microscopy of crystal-violet-stained HME2 parental and post-TGF-β cells following 3 weeks of continuous T-DM1 treatment. **C** The T-DM1-resistant (TDM1R) cells that survived 5 weeks of treatment were further expanded and cultured for a period of 4 weeks in the absence of T-DM1. These cells along with passage-matched parental HME2 cells were subjected 96 h treatments with the indicated concentrations of T-DM1 and assayed for cell viability. Data are the mean ± SE of three independent experiments resulting in the indicated *P* value. **D** Parental, post-TGF-β, and TDM1R HME2 cells were stained with Alexafluor 647-labeled trastuzumab and antibody binding was quantified by flow cytometry. The percentage of cells in each quadrant with reference to forward scatter (FSC) is indicated. Also shown is the mean, ±SD, percentages of low trastuzumab binding (Trastuzumab^Low^) cells of three independent experiments resulting in the indicated *P* values.

Attempts to subculture the T-DM1 recurrent HME2 tumors were unsuccessful, suggesting that these cells had evolved mechanisms of tumor growth that were not present under in vitro culture conditions (Supplementary Fig. 2A). Therefore, we sought to establish a T-DM1 resistant (TDM1R) cell line via prolonged in vitro ADC treatment. However, progressive treatment of HME2 cells with T-DM1 over extended periods of time failed to yield a spontaneously resistant population (Fig. 2A). We recently demonstrated that induction of EMT in the HME2 model is sufficient to facilitate immediate resistance to the ErbB kinase inhibitors, lapatinib and afatinib^18^. In contrast, induction of EMT via pretreatment with TGF-β1 did not induce immediate resistance to T-DM1 (Fig. 2A; 2 weeks). Consistent with the inhibition of microtubules being the mechanism of emtansine, treatment of parental and TGF-β1 pretreated HME2 cells with T-DM1 prevented cell division leading to the formation of non-dividing groups of cells (Fig. 2A, B). Importantly, only those cells that had undergone EMT via pretreatment with TGF-β or spontaneous resistance to lapatinib were capable of giving rise to extremely mesenchymal daughter cells that could resume replication in the continued presence of T-DM1 (Fig. 2A, B and Supplementary Fig. 3). The in vitro TDM1R cell population derived from TGF-β-treated HME2 cells continued to thrive in culture and maintained their mesenchymal phenotype and resistance to T-DM1 even after several passages in the absence of the drug (Fig. 2C and Supplementary Fig. 4). To gain insight into the mechanisms by which induction of EMT facilitates acquisition of resistance to T-DM1, we fluorescently labeled trastuzumab and utilized flow cytometry to quantify changes in drug binding. Consistent with their complete eradication upon T-DM1 treatment, the HME2 parental cells presented as a single population of trastuzumab^+^ cells (Fig. 2A, D). In contrast, induction of EMT with TGF-β clearly produced a distinct population of cells that were resistant to trastuzumab binding, giving rise to a more uniform reduction in trastuzumab binding in the TDM1R cell population (Fig. 2D). These findings suggest that prior induction of cytokine-mediated EMT contributes to diminished trastuzumab binding and is required for acquisition of resistance to T-DM1.

### FGFR1 is sufficient to reduce T-DM1 binding and efficacy

**Fig. 3:**
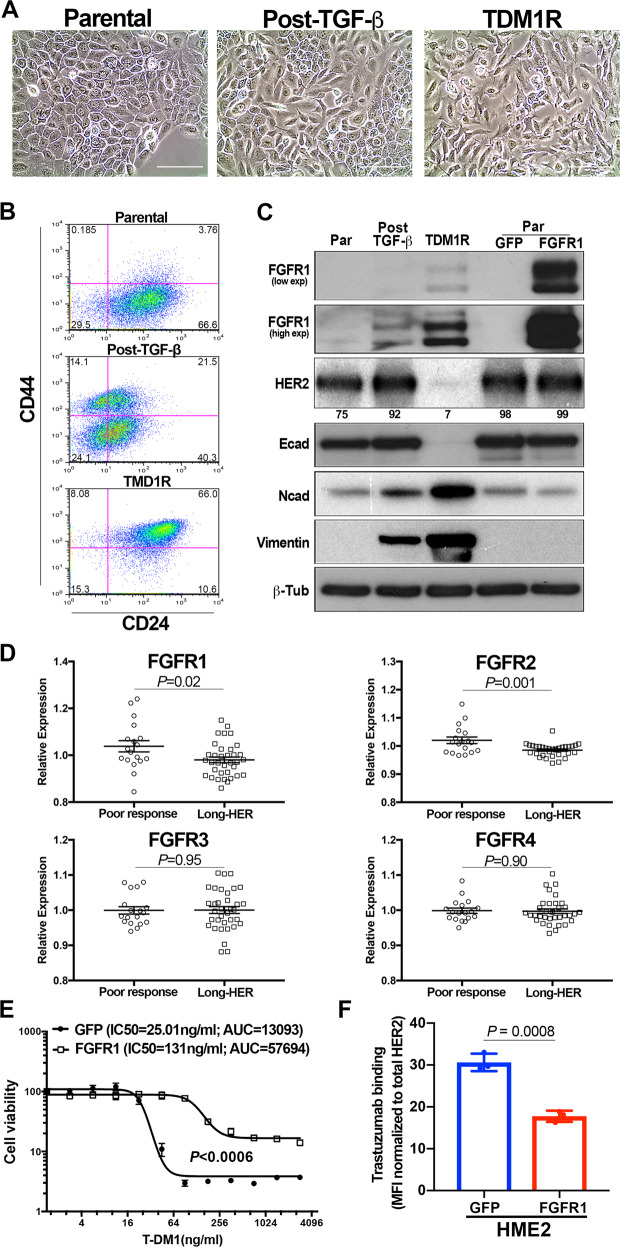
FGFR1 is sufficient to reduce T-DM1 efficacy. **A** Brightfield microscopy of HME2 parental, post-TGF-β, and T-DM1-resistant (TDM1R) cells. **B** The cells described in **A** were analyzed by flow cytometry for cell surface expression of CD24 and CD44. The percentage of cells in each quadrant is indicated. **C** Whole cell lysates from HME2 parental, post-TGF-β, T-DM1-resistant (TDM1R), and HME2 cells constructed to stably express FGFR1 or GFP as a control were analyzed by immunoblot for expression of FGFR1, HER2, E-cadherin (Ecad), N-cadherin (Ncad), vimentin, and β-tubulin (β-Tub) served as a loading control. Data in **B** and **C** are representative of at least three separate analyses. **D** Expression values for FGFR1-4 were analyzed in the Long-HER dataset. Data are the relative expression of individual patients that demonstrated long-term (Long-HER) or short-term (Poor-response) response to trastuzumab treatment, resulting in the indicated *P* values. **E** HME2 cells expressing FGFR1 or GFP as a control were treated with the indicated concentrations of T-DM1 for 96 h at which point cell viability was quantified. Data are normalized to the untreated control cells and are the mean ± SE of three independent experiments resulting in the indicated *P* value. **F** HME2 cells expressing FGFR1 or GFP as a control were incubated with Alexafluor 647-labeled trastuzumab and antibody binding was quantified by flow cytometry. Data are the mean fluorescence intensities, normalized to total HER2 levels as determined by immunoblot, ±SD for three independent experiments resulting in the indicated *P* value.

Our previous studies establish that following TGF-β1 treatment, the purely mesenchymal HME2 culture will asynchronously recover producing a heterogenous population of both epithelial and mesenchymal cells^18^. These morphologically distinct populations can also be readily visualized via flow cytometric analyses for CD44 and CD24 (Fig. 3A and B). Consistent with the stable mesenchymal morphology of the TDM1R cells, they presented as a single population with high levels of CD44, but lacked the diminished expression of CD24 characteristic of TGF-β-induced EMT (Fig. 3A and B)^22^. Other markers of EMT were enhanced upon acquisition of T-DM1 resistance, including loss of E-cadherin and potentiated gains in N-cadherin and vimentin (Fig. 3C). Consistent with the diminished trastuzumab binding observed in Fig. 2, we also observed HER2 expression to be decreased in whole cell lysates from TDM1R cells (Fig. 3C and Supplementary Fig. 3B). To elucidate a mechanistic characterization of potential mediators of T-DM1 resistance, we compared the TDM1R cells to their T-DM1 sensitive, post-TGFβ HME2 counterparts from which they were derived, using kinomic profiling on the PamStation-12 platform. Lysates from TDM1R cells had an increased ability to phosphorylate peptides from FKBP12-rapamycin associated protein (FRAP), a result consistent with enhanced PI3 kinase-mTOR signaling (Supplementary Data 1). Looking upstream to receptors potentially responsible for these events, we observed the autophosphorylation sites of several other ErbB receptors, VEGFRs, and FGFRs to be increased in the TDM1R lysates (Supplementary Data 1).

Upon further investigation into the potential of these receptors in facilitating resistance to T-DM1, we found that the expression level of FGFR1 induced by TGF-β was further enhanced upon acquisition of T-DM1 resistance (Fig. 3C). Although resistance to T-DM1 requires additional mechanisms to overcome the cytotoxic payload, we reasoned that mechanisms of differential trastuzumab binding would be shared between T-DM1 resistance and resistance to unconjugated trastuzumab. As such, we analyzed the Long-HER dataset that compared HER2^+^ patients that experienced a long-term response to trastuzumab with those whose disease progressed within the first year of initiating trastuzumab^24^. This approach indicated that enhanced expression of FGFR1 and FGFR2 is significantly associated with a poor clinical response to trastuzumab (Fig. 3D). Furthermore, analysis of GSE95414 comparing HER2^+^, T-DM1 sensitive, gastric cancer, NCI-N87 cells to their TDM1R counterparts indicated potential gains in the expression of FGFR2, 3, and 4 (Supplementary Fig. S3A). Similarly, analysis of GSE100192 indicated that TDM1R clones derived from the HER2 amplified BT747 breast cancer cell line demonstrate increase in FGFRs or FGF ligands (Supplementary Fig. S3B). Along these lines, separate studies indicate that upon T-DM1 resistance, the BT474 model amplifies the genomic cluster of FGF ligands 3/4/19 (ref. ^16^). To elucidate if FGFR1 is sufficient to provide resistance to T-DM1, we constructed HME2 and BT474 cells to specifically overexpress FGFR1 in the absence of other EMT-associated factors (Fig. 3C and Supplementary Fig. S4; ref. ^18^). Using this approach, we found that overexpression of FGFR1, when in the presence of exogenous ligand, was sufficient to significantly reduce the dose response to T-DM1 (Fig. 3E and Supplementary Fig. S5). Unlike the TDM1R cell line, we did not detect an appreciable decrease in HER2 expression upon directed overexpression of FGFR1 (Fig. 3C). However, FGFR1 overexpression was sufficient to cause a significant reduction in trastuzumab binding as determined by flow cytometry, and this could not be rescued by treatment with FIIN4, a covalent inhibitor of FGFR kinase activity (Fig. 3F and Supplementary Fig. S6).

### FGFR1 increases tumor recurrence following T-DM1-induced MRD

**Fig. 4:**
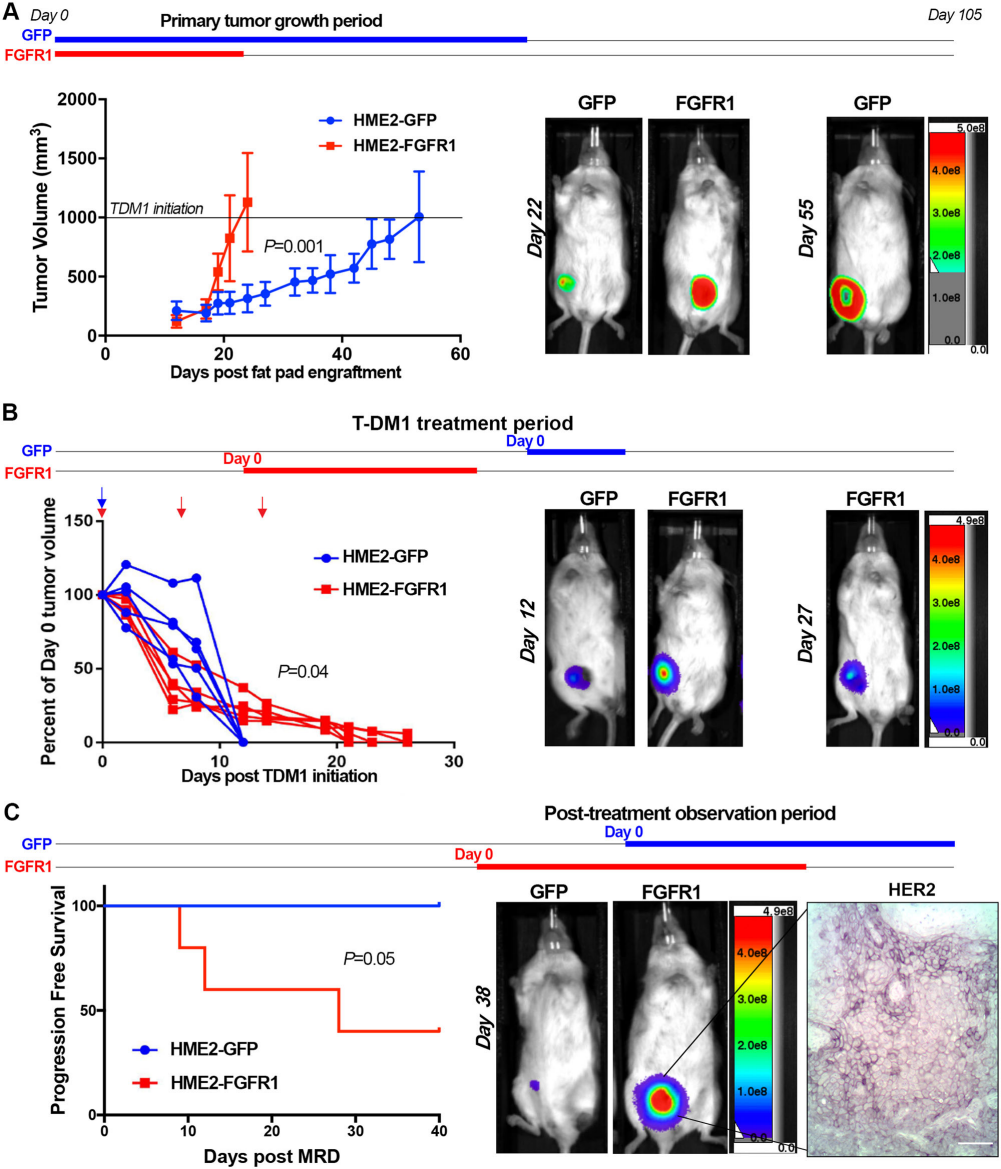
FGFR1 increases tumor recurrence following T-DM1-induced minimal residual disease. **A** Time line demarking length of the entire experiment shown in this figure, the primary tumor growth period for HME2 cells constructed to express FGFR1 or GFP as a control are highlighted in blue and red, respectively. Cells were engrafted onto the mammary fat pad of female NRG mice (2 × 10^6^ cells/mouse; *n* = 5 mice per group). Tumor growth was monitored via digital caliper measurements at the indicated time points. T-DM1 treatment was initiated in each group when tumors reached an average of 1000 mm^3^, horizontal line. Representative bioluminescent images for each group are shown at the indicated time points. Days are in reference to tumor engraftment (Day 0). **B** Time line demarking the T-DM1 treatment periods for the GFP (blue line) and FGFR1 expressing groups (red line). T-DM1 was administered via I.V. injections (9 mg/kg) at day 0 for HME2-GFP tumors and days 0, 7, and 14 for HME2-FGFR1 tumors (arrows). Tumor regression was monitored via digital caliper measurements at the indicated time points. Representative bioluminescent images for each group are shown at the indicated time points. Days are in reference to initiation of T-DM1 treatment (day 0) for each group. **C** Time line demarking the post-treatment observation period for the GFP (blue line) and FGFR1 (red line) expressing groups. Once tumors regressed to a non-palpable state of minimal residual disease (MRD), mice were left untreated and monitored for tumor recurrence via digital caliper measurements. Representative bioluminescent images for each group are shown at the indicated time points. Days are in reference to achievement of MRD (day 0) for each group. A representative HER2 IHC is shown for the recurrent tumors. Survival data in **C** were analyzed via a log rank test where tumor recurrence of >50 mm^3^ was set as a criteria for disease progression. Data in **A** are the mean ± SD of five mice per group resulting in the indicated *P* value. In **B**, tumor size for each mouse is plotted individually. Data in **A** and **B** were analyzed via a two-way ANOVA.

We next sought to evaluate the impact of FGFR1 expression on HME2 tumor growth and response to T-DM1. Overexpression of FGFR1 promoted a significant increase in growth rate of HME2 tumors upon mammary fat pad engraftment, leading to differential TDM1 treatment initiation times for matched tumor sizes (Fig. 4A). Irrespective of FGFR1 expression, the liquid-filled masses characteristic of large HME2 tumors quickly became necrotic after a single dose of TDM1 (Fig. 4B). However, following this initial rapid reduction in tumor size, only the FGFR1 overexpressing tumors maintained a more solid mass which required two additional rounds of T-DM1 treatment to achieve complete tumor regression (Fig. 4B). As we observed in Fig. 1, the MRD associated with these nonpalpable lesions could still be detected by bioluminescence (Fig. 4B). Following achievement of T-DM1-induced MRD, none of the control tumors progressed within the 40-day post-treatment observation period (Fig. 4C). In contrast, over 50% of the FGFR1 overexpressing tumors underwent disease progression during this same post-treatment time frame (Fig. 4C). In contrast to the marked loss of HER2 expression in the spontaneously resistant HME2 lesions noted in Fig. 1, the FGFR1 overexpressing recurrent tumors were capable of maintaining heterogeneous HER2 expression (Fig. 4C). Taken together, these data strongly suggest that enhanced expression of FGFR1 inhibits T-DM1 binding, facilitating therapeutic persistence of HER2^+^ cells and post-treatment tumor recurrence.

### TDM1R cells are sensitive to covalent inhibition of FGFR

**Fig. 5:**
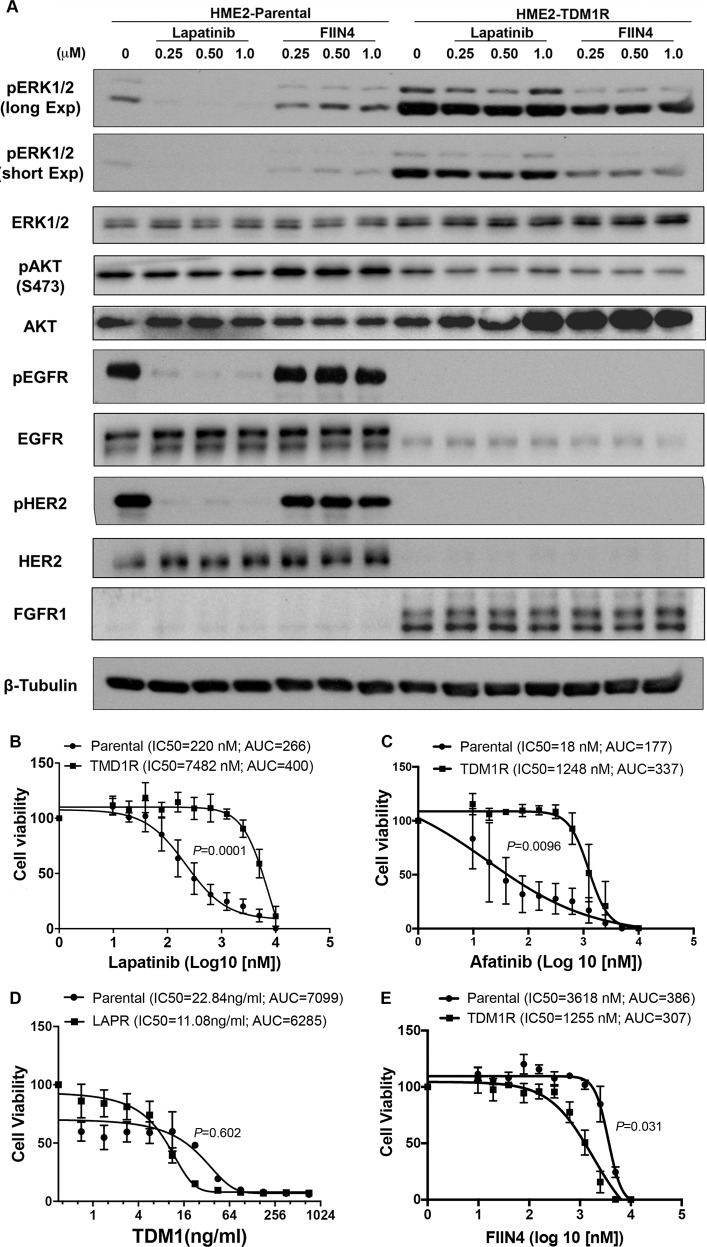
T-DM1-resistant cells are sensitive to covalent inhibition of FGFR. **A** HME2 parental and T-DM1-resistant (TDM1R) cells were treated with the indicated concentrations of lapatinib or FIIN4 for 2 h. Cell lysates were subsequently assayed by immunoblot for phosphorylation of ERK1/2, HER2, AKT, and EGFR. Total levels of ERK1/2, AKT, EGFR, HER2, and FGFR were also assessed. β-tubulin (β-Tub) served as a loading control. Data in **A** are representative of at least two independent experiments. **B** HME2 parental cells (parental) and TDM1R cells were plated in the presence of the indicated concentrations of lapatinib for 96 h at which point cell viability was determined. **C** HME2 parental and TDM1R cells were plated in the presence of the indicated concentrations of afatinib for 96 h at which point cell viability was determined. **D** HME2 parental and lapatinib-resistant (LAPR) cells were plated in the presence of the indicated concentrations of T-DM1 for 96 h at which point cell viability was determined. **E** HME2 parental and TDM1R cells were plated in the presence of the indicated concentrations of FIIN4 for 96 h at which point cell viability was determined. Data in **B**–**E** are the mean ± SE of at least three independent experiments resulting in the indicated *P* values.

Given the changes in ErbB kinase signaling observed in the TDM1R cells and the ability of FGFR overexpression to facilitate recurrence following ADC therapy, we next sought to evaluate the ability of specific kinase inhibitors to target TDM1R cells as compared to their T-DM1 sensitive counterparts. Lapatinib is a clinically used kinase inhibitor that targets both EGFR and HER2, and we recently developed FIIN4, a covalent kinase inhibitor that targets FGFR1-4 (ref. ^19^). Treatment of the HME2 parental cells with lapatinib led a robust inhibition of HER2 phosphorylation and downstream blockade of ERK1/2 phosphorylation (Fig. 5A). Consistent with the reduced expression of total HER2, phosphorylated levels of HER2 were undetectable in the TDM1R cells and ERK1/2 phosphorylation was minimally inhibited by lapatinib (Fig. 5A). In contrast, treatment of the HME2 parental cells with FIIN4 had no effect on HER2 or ERK1/2 phosphorylation, but FIIN4 markedly diminished FGFR and ERK1/2 phosphorylation in the TDM1R cells (Fig. 5A and Supplementary Fig. 7). Importantly, TDM1R cells also demonstrated robust resistance to lapatinib, even thought they had never been exposed to this compound previously (Fig. 5B). Similarly, TDM1R cells were also resistant to afatinib, a more potent second-generation covalent kinase inhibitor capable of targeting EGFR, HER2, and ErbB4 (Fig. 5C; ref. ^25^). In contrast, HME2 cells that had previously been selected for resistance to lapatinib (LAPR) maintained expression of HER2 and were similarly sensitive to T-DM1 as compared to the HME2 parental cells (Fig. 5D; ref. ^18^). TDM1R cells were significantly more sensitive to FIIN4 as compared to the HME2 parental cells (Fig. 5E). Finally, combined treatment of T-DM1 and FIIN4 in FGFR1 overexpressing HME2 cells led to a significant increase in growth inhibition as compared to each single agent (Supplementary Fig. 8). Taken together these data indicate that resistance to HER2-targeted ADC therapy predicates acquisition of resistance to ErbB-targeted kinase inhibitors but the reverse is not true. Importantly, these drug-resistant populations become increasingly sensitive to covalent inhibition of FGFR.

### TDM1R tumors respond to systemic inhibition of FGFR

**Fig. 6:**
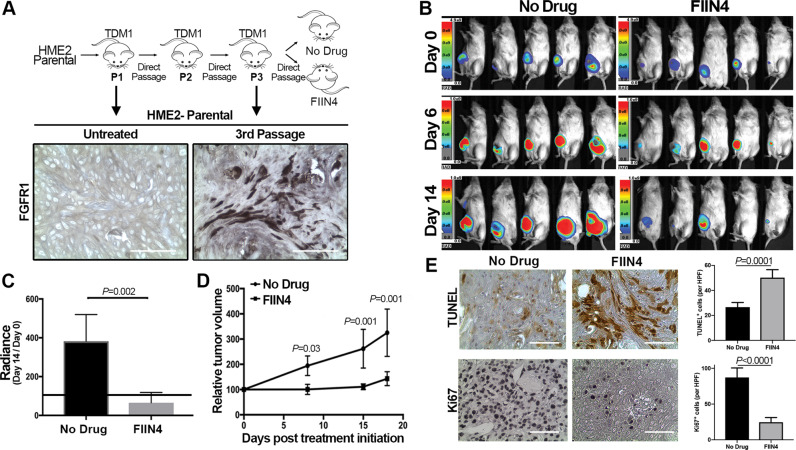
T-DM1-resistant tumors respond to systemic inhibition of FGFR. **A** Schematic representation of the in vivo derivation of T-DM1-resistant HME2 tumors. HME2 parental cells (2 × 10^6^) were engrafted onto the mammary fat pad of an NSG mouse. This mouse was treated with T-DM1 until tumor regression was observed. Sections of the remaining tumor were directly transferred onto recipient mice. This was repeated twice until transferred tumors that no longer responded to T-DM1 therapy were identified. At this point, sections of a T-DM1-resistant tumor were assessed by IHC for FGFR1 expression as compared to the originally engraphed HME2 tumors. Representative FGFR1 IHC staining is shown. **B** Bioluminescent imaging of mice bearing the T-DM1-resistant tumors described in **A**. These mice were left untreated (No Drug) or were treated with FIIN4 (100 mg/kg/q.o.d.). **C** Bioluminescent quantification of control and FIIN4-treated animals bearing T-DM1-resistant tumors. Data are normalized to the tumor luminescence values at the initiation of FIIN4 treatment (day 0). **D** Tumor size as determined by digital caliper measurements at the indicated time points during FIIN4 treatment. For **C** and **D**, data are the mean ± SE of five mice per group resulting in the indicated *P*-values. **E** Representative Ki67 and TUNEL staining of P3, T-DM1-resistant tumors from untreated and FIIN4-treated groups. Also shown are the mean ± SD of TUNEL and Ki67-positive cells per high powered field (HPF), *n* = 5, resulting in the indicated *P* values.

We next sought to validate our in vitro findings by evaluating the efficacy of FGFR inhibition in the treatment of tumors that had acquired in vivo resistance to T-DM1. To do this, we treated HME2 tumor-bearing mice with T-DM1 and prior to complete MRD, sections of the tumors were directly passaged onto additional mice. This process was repeated twice until we obtained a cohort of mice with growing tumors that did not respond to T-DM1 (Fig. 6A). Similar to what was observed in tumors that were recovered following induction of MRD and in our in vitro TDM1R cells, these serially passaged in vivo-derived TDM1R tumors also demonstrated a diminution in HER2 expression and modulated markers of EMT, including enhanced FGFR1, as compared to untreated HME2 tumors (Fig. 6A and Supplementary Fig. 8). Importantly, treatment with FIIN4 was capable of significantly inhibiting the growth of these T-DM1-resistant tumors (Fig. 6B–D). This inhibition of tumor growth was consistent with induction of apoptosis and decreased proliferation as visualized by TUNEL and Ki67 staining in tumors from FIIN4-treated mice as compared to untreated controls (Fig. 6E).

**Fig. 7:**
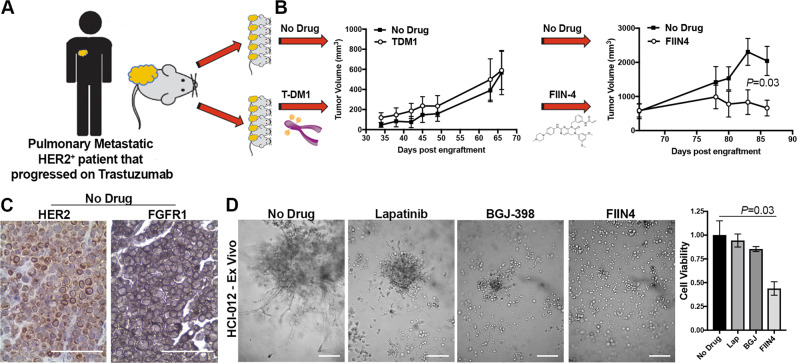
Trastuzumab-resistant patient-derived xenografts are sensitive to covalent inhibition of FGFR. **A** Schematic representation of the expansion protocol of HCI-012. **B** Tumor-bearing mice were split into two cohorts consisting of an untreated group and a group that was initially treated with T-DM1 (5 mg/kg). Due to the lack of response to TDM1, these animals were switched to FIIN4 (25 mg/kg/p.o.d). Tumor size was measured by digital caliper measurements at the indicated time points. Data are the mean ± SE of 5 mice per group resulting in the indicated *P* value. **C** Representative histological sections of untreated HCI-012 tumors stained with antibodies for HER2 and FGFR1. **D** Ex vivo HCI-012 tumor cells were grown for 20 days under 3D culture conditions in the presence or absence of the indicated compounds. Representative images are shown and cell viability was quantified by cell titer glow. Data are mean ± SD of triplicate wells treated with the indicated compounds.

Next, we utilized a patient-derived xenograft (PDX), HCI-012, that was isolated from a pleural effusion of a patient originally diagnosed with a HER2^+^ primary tumor (Fig. 7A). This patient’s disease progressed while on trastuzumab therapy (Fig. 7A). Consistent with this clinical failure of trastuzumab, these PDX tumors displayed variable and non-membranous staining for HER2 and mice bearing these PDX tumors failed to respond to T-DM1 treatment (Supplementary Fig. 9 and Fig. 7B, C). These PDX tumors also demonstrated readily detectable staining for FGFR1 (Fig. 7C). Clinically, lapatinib is indicated as a second line therapy in HER2^+^ patients that do not respond to trastuzumab. Treatment with lapatinib did blunt the 3D invasive phenotype of the HCI-012 PDX when cultured under 3D ex vivo conditions. However, consistent with our data from Fig. 5, the overall growth of these T-DM1-resistant PDX ex vivo cultures was not inhibited by lapatinib treatment (Fig. 7D). In contrast, treatment of these ex vivo cultures or tumor-bearing mice with FIIN4 led to significant inhibition of tumor growth (Fig. 7B, D). Consistent with our previous studies, FIIN4 also demonstrated enhanced potency as compared to an identical concentration of its ATP competitive structural analog, BGJ-398 (Fig. 7D). Taken together these data indicate that TDM1R tumors can be effectively targeted via covalent inhibition of FGFR kinase activity.

## Discussion

Genomic amplification and high-level protein expression of HER2 cause constitutive oncogenic activity in a significant subset of breast cancer patients. These molecular events have precipitated robust diagnostics for HER2-targeted therapeutics in these patients. Following disease progression on trastuzumab/pertuzumab, HER2^+^ patients can be treated with T-DM1, trastuzumab-deruxtecan, lapatinib, neratinib, tucatinib, or various combinations thereof. These therapies are predicated on the assumption that tumors and their corresponding metastases will express extracellular portions of HER2 and remain addicted to HER2 or other ErbB receptors for growth. However, increasing numbers of experimental and clinical studies indicate that following treatment with HER2-targeted therapies tumors can activate a variety of mechanisms to resist antibody binding and eject the cytotoxic payload of ADCs, leading to tumor cell persistence. Herein, using an exogenous expression system, we observed downregulation of HER2 upon acquisition of resistance to T-DM1. While discordance in HER2 expression following treatment is not a common occurrence in breast cancer, it has been observed clinically^26^. Given our use of an exogenous expression model and immunodeficient mice, our findings suggest that acquisition of HER2 discordance is an active process that does not require immune-mediated cell clearance or endogenous transcriptional elements. Overall, the concept of HER2-initated tumors being capable of undergoing recurrence in a HER2-independent, EMT-driven fashion is supported by recent studies using doxycycline regulated models of HER2 expression^27,28^.

For tumor recurrence and disease progression to occur, tumors cells must not only persist during T-DM1 treatment, but they must also engage an alternate proliferative program. Herein, we demonstrate that enhanced expression of FGFR1 is sufficient to drive tumor recurrence following induction of MRD by T-DM1. Previous studies from our lab and others suggest that FGFR can act as a bypass mechanism to facilitate immediate resistance to ErbB kinase inhibitors^15,16,18,29^. Furthermore, FGFR signaling has also been identified as a mechanism of resistance to endocrine therapies in breast and prostate cancer^14^. Therefore, FGFR appears to constitute a critical node in acquisition of drug resistance. The reasons for this are potentially numerous, but a possible explanation is the inducible nature of FGFR expression. In particular, FGFR1, FGFR3, and FGF2 expressions are dramatically upregulated during the processes of EMT^15,18^. Our previous studies demonstrate the acquired resistance to lapatinib or directed overexpression of the EMT transcription factor Twist are capable to inducing FGFR1, but the precise mechanisms of FGFR1 upregulation during EMT remain to be fully elucidated^18,19^. In any event, enhanced FGFR signaling presents a functional and targetable link between EMT and the acquisition of drug resistance^30^.

FGFR signaling is clearly capable of acting as a bypass pathway during the acquisition of resistance to ErbB kinase inhibitors. However, our current data suggest that enhanced FGFR1 expression also plays a more active role in manifesting resistance to HER2 antibody therapies by disrupting trastuzumab’s ability to bind to HER2. Determining the mechanisms by which FGFR prevents trastuzumab binding is currently under investigation in our laboratory. If FGFR physically disrupts antibody binding, than a proteolysis targeting chimera (PROTAC) strategy may be required to degrade FGFR, reestablish trastuzumab binding, and prevent TDM1 persistence. In contrast, FGFR signaling has been shown to enhance the expression of ADAM10, a matrixmetaloproteinase capable of diminishing cellular binding of trastuzumab via its cleavage of the extracellular portion HER2^31^. Our studies do not rule out this kind of indirect mechanism of reduced trastuzumab binding. Our data do clearly show that diminished binding of trastuzumab can manifest upon induction of EMT or by direct overexpress of FGFR1, independent of other EMT events. Overall, our data are consistent with a model in which cytokine-induced EMT upregulates FGFR1 which can immediately serve as a bypass pathway to overcome inhibition of ErbB receptor kinase activity. In contrast, a TDM1R population does not immediately emerge in TGF-β pretreated cultures, but induction of EMT allows for the persistence of a subpopulation of cells in the face of T-DM1 treatment. This drug persistent subpopulation is eventually able to give rise to a proliferative population that is driven by FGFR signaling and is fully resistant to T-DM1. This presents a unique combination of both subpopulation selection and phenotype plasticity, two processes typically thought to be mutually exclusive in drug resistance. Our studies suggest covalent inhibition of FGFR as a potential approach for targeting T-DM1 resistance tumors. We developed FIIN4 as the first-in-class covalent inhibitor of FGFR, and an additional covalent FGFR inhibitor, TAS-120, is currently in phase 2 clinical trials in patients with advanced solid tumors (NCT02052778)^19^. These studies and the studies herein demonstrate the enhanced efficacy of covalent kinase inhibition of FGFR as compared to ATP competitive inhibitors. This may be a result of the structural stabilization of the inactivate confirmation that results upon covalent engagement of FGFR^32^. However, erdafitinib and pemigatinib, extremely potent competitive inhibitors of FGFR, have recently been FDA approved. Therefore, clinical investigation of sequential and/or direct combinations of FGFR inhibitors with T-DM1 in the HER2^+^ setting is possible and clearly warranted.

## Methods

### Cell culture and reagents

HMLE cells were a kind gift from Sendurai Mani (MD Anderson Cancer Center). These cells were constructed to stably express firefly luciferase via lentiviral transduction and selection with blasticidine. HER2-transformed HMLE cells (HME2) were constructed via lentiviral transduction of pBabe (addgene #40978) and stable selection using puromycin. Once transformed by HER2, HME2 cells are cultured in DMEM containing 10% FBS and 10 μg/ml of insulin. BT474 cells were obtained from the ATCC. HME2 and BT474 cells stably overexpressing FGFR1 were constructed by lentiviral transduction and stable selection using hygromycin as previously described^18^. Trastuzumab and trastuzumab emtansine (T-DM1) were obtained from Genentech through the material transfer agreement program. Where indicated, HME2 cells were treated with TGF-β1 (5 ng/ml) every 3 days for a period of 4 weeks to induce EMT. These EMT-induced HME2 cells were further treated with T-DM1 (250 ng/ml) every 3 days until resistant colonies emerged, these cells were pooled and cultured as the TDM1R population. Cells were validated for lack of mycoplasma contamination using the IDEXX Impact III testing on July 24, 2018.

### Xenograft studies and drug treatments

HME2 cells (2 × 10^6^) expressing firefly luciferase were injected into the duct of the fourth mammary fat pad of female NRG mice. When tumors reached a size of 200 mm^3^, mice were treated with the indicated concentrations of T-DM1 via tail vein injection. Presence of tumor tissue was visualized by bioluminescence imaging following I.P. administration of luciferin (Gold Bio). Where indicated, viable pieces of HME2 tumor tissue were directly transplanted into the exposed fat pad of recipient NRG mice. Similarly, pieces of human-derived HCI-012 PDX (Huntsman Preclinical Research Resource) were engrafted onto the exposed mammary fat pad of female NRG mice. Tumor bearing mice were treated with T-DM1 as indicated followed by FIIN4 (25 mg/kg/q.o.d) resuspended in DMSO and then further diluted in a solution 0.5% carboxymethyl cellulose and 0.25% Tween-80 to a final concentration of 10% DMSO, for administration to animals via oral gavage. Mammary tumor sizes were measured using digital calipers and the following equation was used to approximate tumor volume: *V* = (length^2^) × (width) × (0.5). All animal experiments were conducted under IACUC approval from Purdue University.

### Cell biological assays

For ex vivo 3D culture, viable human PDX tissues were dissected as above but instead of transfer onto recipient animals, pieces were further mechanically dissected and treated with trypsin-EDTA. These cells were shaken several times and incubated at 37 °C. Cells were then filtered through a 50-μM filter and plated onto a 50-μl bed of growth factor reduced cultrex (Trevigen) in a white-walled 96-well plate. These cultures were allowed to grow for 20 days in the presence or absence of the indicated kinase inhibitors (1 μM). Two-dimensional cell growth dose response assays were conducted in white-walled 96-well plates. Cells (5000 cells/well) were plated in the presence of the indicated concentrations of T-DM1 or kinase inhibitors and cultured for 96 h. In both cases, cell viability was determined by Cell Titer Glo assay (Promega).

### Immuno-assays

For immunoblot assays, lysates were generated using a modified RIPA buffer containing 50 mM Tris pH 7.4, 150 mM NaCl, 0.25% sodium deoxycholate, 0.1% SDS, 1.0% NP-40, containing protease inhibitor cocktail (Sigma), 10 mM sodium orthovanadate, 40 mM β-glycerolphosphate, and 20 mM sodium fluoride. Following SDS PAGE and transfer, PVDF membranes were probed with 1:1000 dilutions of antibodies specific for pERK1/2 (9101), pHER2 (2243), pAKT (4060), pEGFR (6963), AKT (4685), EGFR (4267), ERR1/2 (4695), FGFR1 (9740), HER2 (4290; Cell signaling Technologies), E-cadherin (610182), N-cadherin (610920), Vimentin (550513; BD biosciences), or β-Tubulin (E7-s; Developmental Studies Hybridoma Bank). All blots shown together were derived from the same experiment and processed in parallel. For immunocytochemistry, formalin-fixed paraffin-embedded tissue sections were deparaffinized and stained with 1:50 dilutions of antibodies specific for HER2 (4290), FGFR1 (HPA056402; Sigma), Ki67 (550609; BD biosciences) or were processed using the TUNEL Assay Kit (ab206386). Additionally, cells were trypsinized and incubated with antibodies specific for FITC conjugated CD44 (338804) and PerCP conjugated CD24 (311113; Biolegend) or trastuzumab conjugated with Alexafluor 647 according to the manufacturer’s instructions (A20181; Thermo Scientific). Following antibody staining, these cells were washed and analyzed by flow cytometry.

### Kinomic analyses

Lysates from HME2 cell conditions indicated above, were analyzed on tyrosine chip (PTK) and serine–threonine chip (STK) arrays using 15 μg (PTK) or 2 μg (STK) of input material as per standard protocol in the UAB Kinome Core as previously described^23,33^. Three replicates of chip-paired samples were used and phosphorylation data were collected over multiple computer controlled kinetic pumping cycles, and exposure times (0, 10, 20, 50, 100, 200 ms) for each of the phosphorylatable substrates. Slopes of exposure values were calculated, log2 transformed, and used for comparison. Raw image analysis was conducted using Evolve2, with comparative analysis done in BioNavigator v6.2 (PamGene, The Netherlands).

### Statistical analyses

Data from the Long-HER study used to support the findings of this study have been deposited in GEO with the GSE44272 accession code. A summary of the patient characteristics and description of the ethics approvals can be found in the original publication of these data^24^. Expression values of FGFR1-4 were obtained from Affymetrix probes, 11747417_x_at, 11740159_x_at, 11717969_a_at, 11762799_a_at, respectively. Expression values were normalized to the average probe value for the entire group and differences between the long-term responders (Long-HER) and control (Poor response) groups were compared via an two-sided, unpaired *T*-test. Two-way ANOVA or two-sided *T*-tests were used where the data met the assumptions of these tests and the variance was similar between the two groups being compared. No exclusion criteria were utilized in these studies. A Log-rank test was performed to calculate statistically significant differences in disease-free survival of HME2-GFP and HME2-FGFR1 tumor–bearing mice. *P* values for all experiments are indicated, values of <0.05 were considered significant. A preliminary version of this manuscript has been published as a preprint^34^.

### Reporting summary

Further information on experimental design is available in the Nature Research Reporting Summary linked to this paper.

## Supplementary information

Supplemental Material

Supplementary Data 1

Reporting Summary Checklist

## Data Availability

The datasets generated and analyzed during the current study are available from the corresponding author, Dr. Michael Wendt (email address: mwendt@purdue.edu), upon reasonable request, as described in the following figshare metadata record: 10.6084/m9.figshare.13148360^35^. Kinome data are publicly available in Supplementary Data 1. Uncropped Western blots are available as part of the supplementary information files. The Long-HER dataset analyzed during the study is publicly available in Gene Expression Omnibus: https://identifiers.org/geo:GSE44272^36^.

## Code availability

No custom code was used to generate the data contained in this manuscript.
