# Supplementary material for: Fibroblast growth factor receptor facilitates recurrence of minimal residual disease following trastuzumab emtansine therapy
Source: NPJ Breast Cancer. 2021 Jan 21;7:5. doi: 10.1038/s41523-020-00213-5 (PMC7820437; doi:10.1038/s41523-020-00213-5)
Supplement: Supplementary file 1 — Supplemental Material [file 41523_2020_213_MOESM1_ESM.pdf]

Fibroblast growth factor receptor facilitates recurrence of minimal residual disease following trastuzumab emtansine therapy

Saeed S. Akhand[1], Hao Chen[1], Stephen Connor Purdy[1], Zian Liu[1], Joshua C. Anderson[2], Christopher D. Willey[2], Michael K. Wendt[1*]

Supplementary Information:

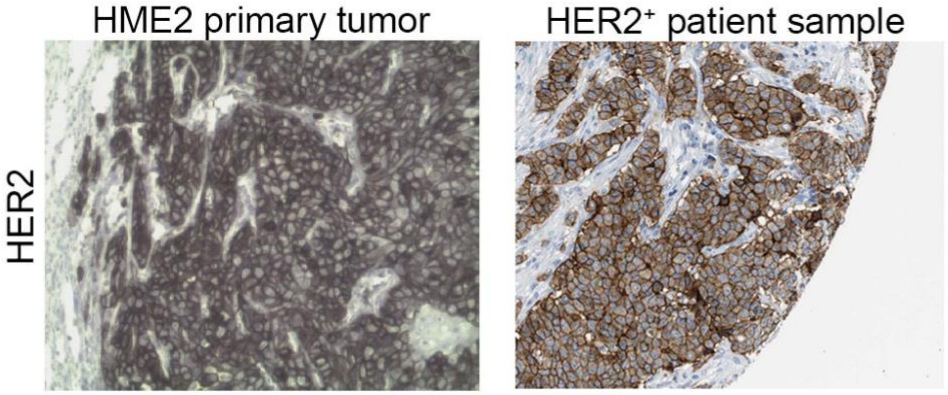

Supplementary Figure 1. HER2 expression in the HME2 tumors is consistent with human disease. (left frame) IHC analysis for HER2 expression in untreated HME2 mammary fat pad tumors. (right frame) IHC analysis for HER2 expression in a HER2+ patient primary tumor biopsy. The patient tumor image was obtained from the Human Protein Atlas (www.proteinatlas.org).

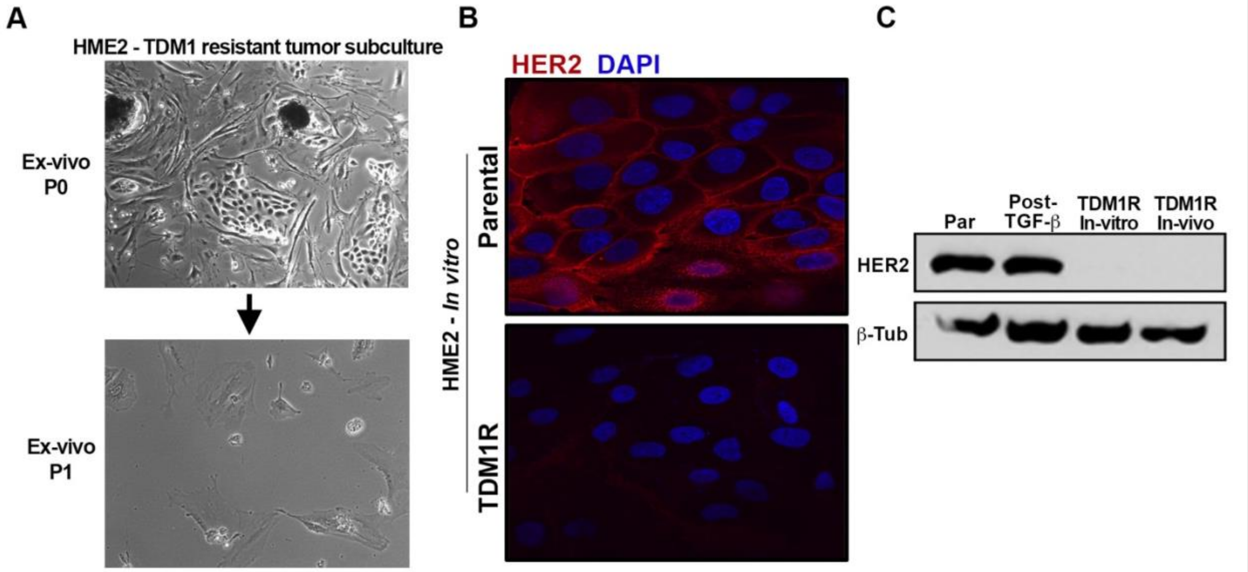

Supplementary Figure 2. HER2 expression is diminished upon acquisition of resistance to T-DM1. **A** *Ex-vivo* subculture of HME2 tumors that recurred after T-DM1-induced minimal residual disease as shown in Figure 1. These cells failed to thrive in culture. **B** HME2 parental cells and their *in vitro*-derived T-DM1 resistant (TDM1R) counterparts were fixed, permeabilized and stained for HER2. These cells were counter stained with DAPI to visualize the nucleus. **C** HME2 parental cells (Par), those treated and recovered from TGF-β1 (Post-TGF-β), those treated and recovered from TGF-β1 and subsequently selected for by continuous treatment with T-DM1 (TDM1R *In-vitro*), and primary culture from P3 HME2 tumors selected for resistance to T-DM1, as described in Figure 7a of the main text (TDM1R *In-vivo*), were assayed by immunoblot for expression of HER2 and β-tubulin (β-Tub) served as a loading control.

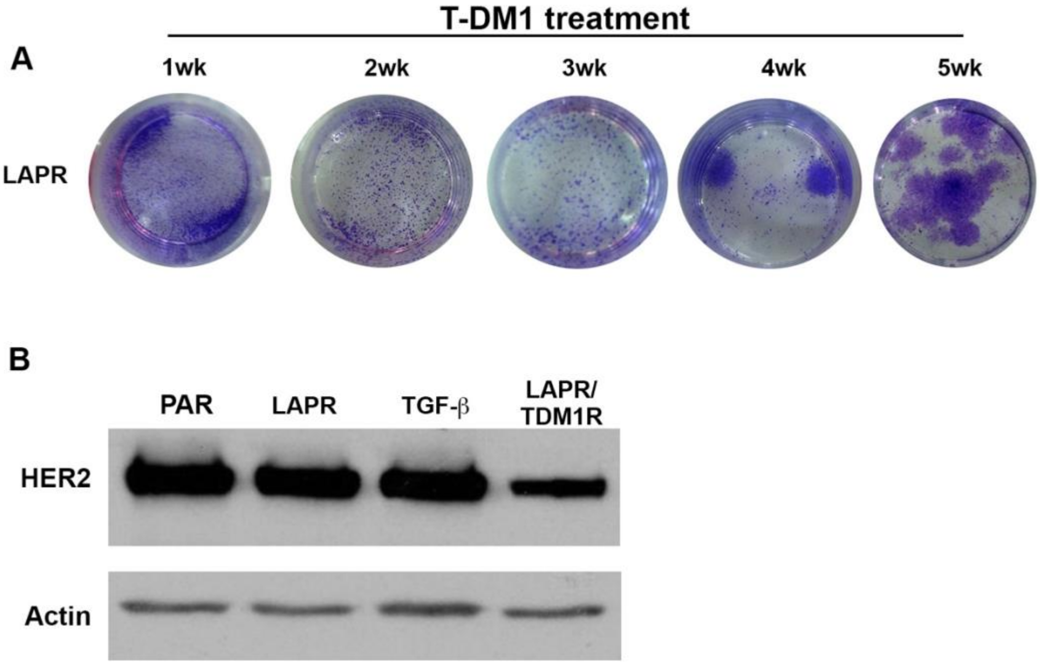

Supplementary Figure 3. Lapatinib induced EMT facilitates acquisition of resistance to T-DM1. HME2 cells were selected for resistance to lapatinib via prolonged culture in the presence of the drug. **A** These lapatinib resistant (LAPR), mesenchymal cells were subsequently treated with T-DM1 for indicated amounts of time and representative culture wells were stained with crystal violet to visualize emergence of drug resistant colonies. **B** Expression of HER2 was analyzed by immunoblot in parental (PAR), LAPR, TGF-β treated, and the T-DM1 resistant cells derived from the LAPR population.

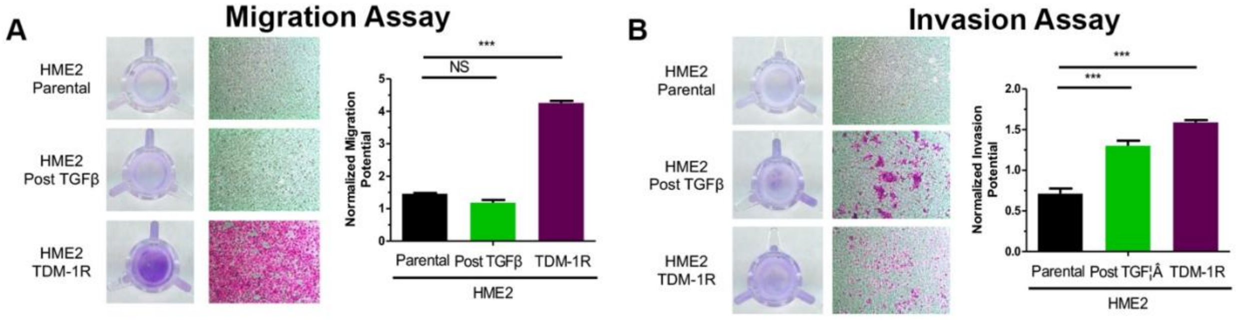

Supplementary Figure 4. Increased migration and invasion upon acquisition of resistance to T-DM1. A and B HME2 parental cells, those treated with TGF-β1, and the TDM1R cells were placed in the top well of a transwell assay in serum free media and 10% serum was placed in the bottom well. After 18 hours (A) or 48 hours (B) cell were removed from the top well and migrated cells on the bottom of the well were stained with crystal violet and enumerated.

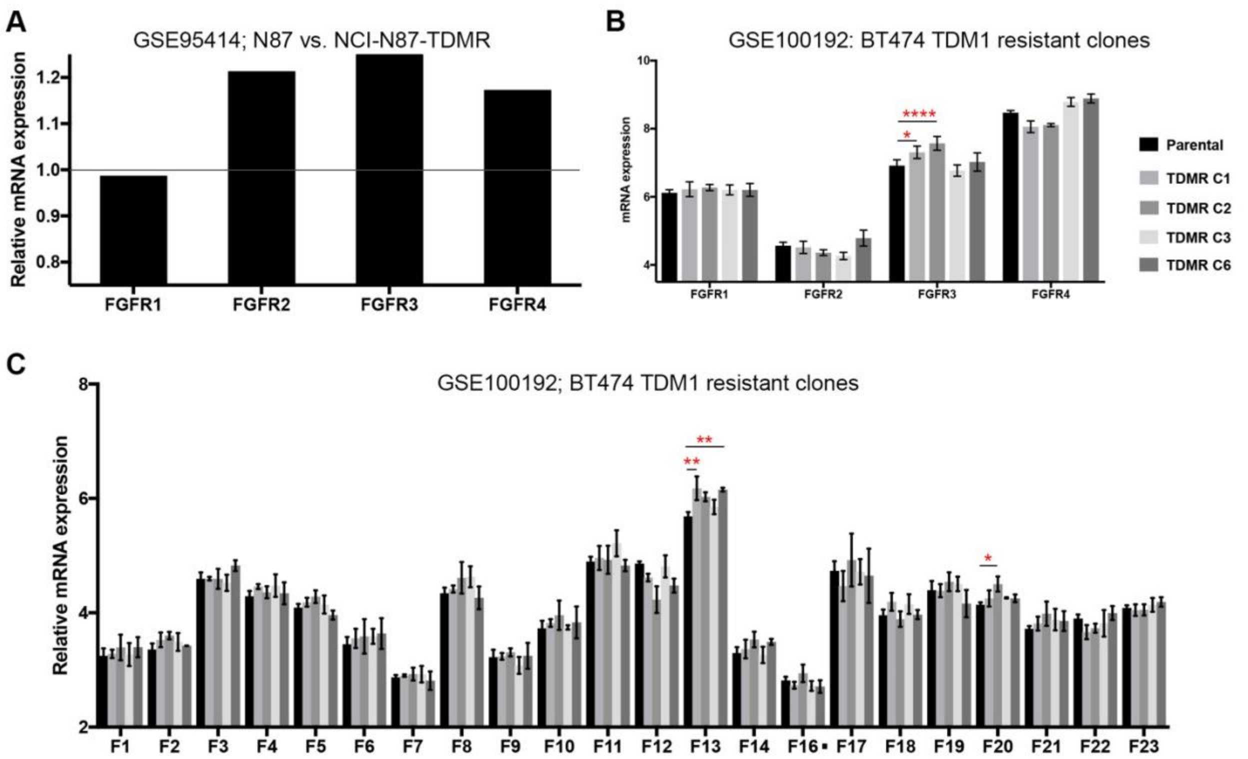

Supplementary Figure 5. Enhanced FGFR expression upon acquisition of resistance to TDM1. **A** Expression levels of FGFR1-4 in the NCI-N87 cells selected for resistant to TDM1. Data are extracted from a single RNA sequencing experiment and are normalized to the untreated control cells. **B and C** Expression levels of FGFR1-4 (B) and FGF 1-23 (C) in four different TDM1 resistant BT474 clones (C1-3 and C6). Data are mean ±SD of expression values from triplicate RNA sequencing experiments conducted for the parental cells and each TDM1 resistant clone. Resulting the indicated P values.

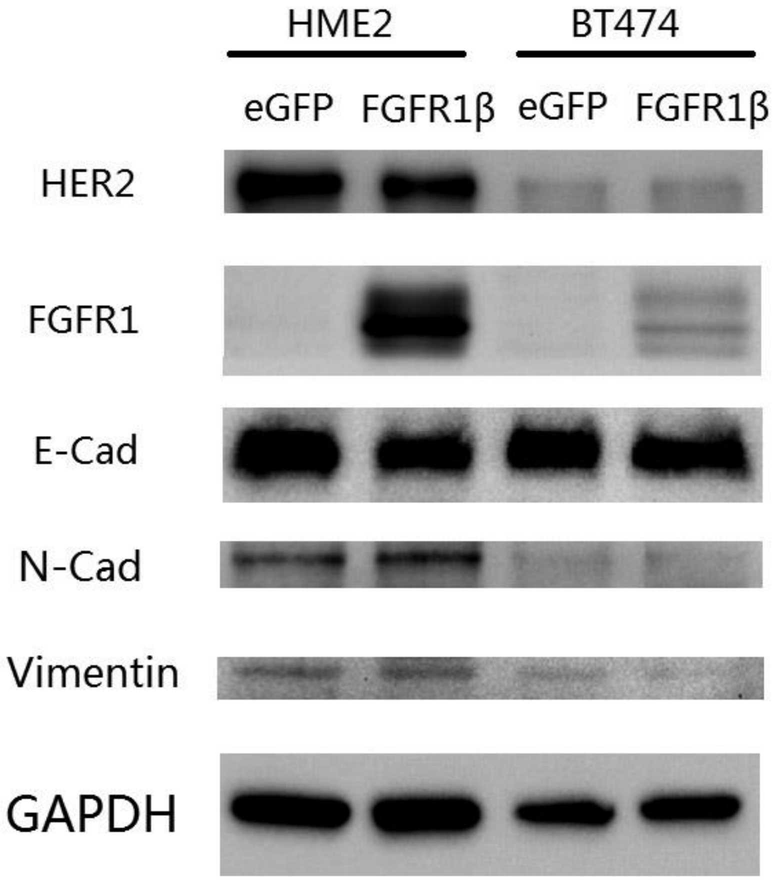

Supplementary Figure 6. Overexpression of FGFR1 does not induced EMT. HME2 and BT474 cells were constructed to stably overexpress FGFR1 or eGFP as a control. FGFR1 and other markers of EMT, E-cadherin (E-cad), N-cadherin (N-cad), and vimentin were analyzed by immunoblot. Expression of GAPDH served as a loading control.

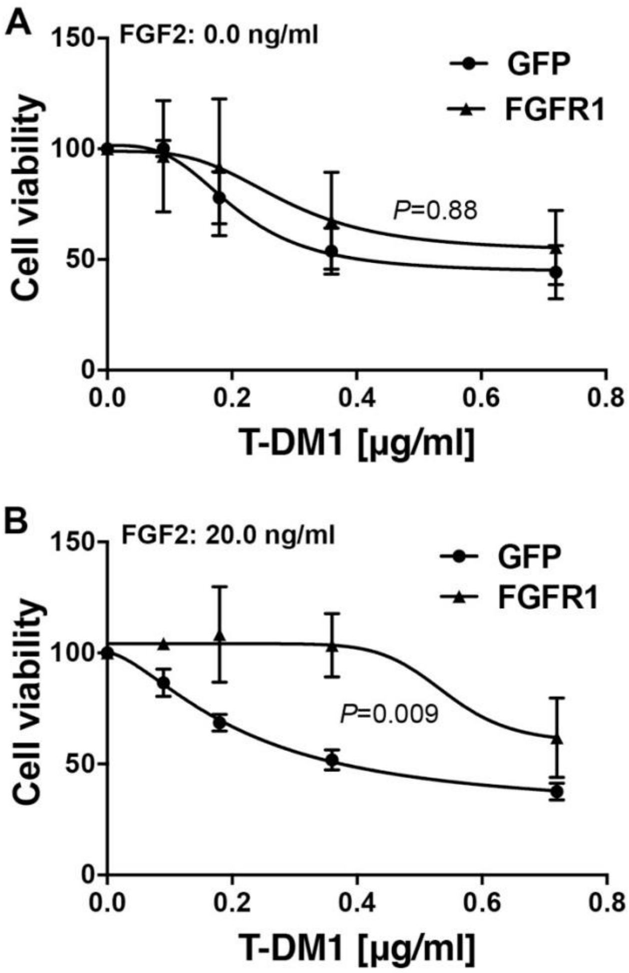

Supplementary Figure 7. FGFR signaling is sufficient to diminish BT474 response to T-DM1. **A and B,** HER2 amplified BT474 cells expressing FGFR1 or GFP as a control were treated with the indicated concentrations of T-DM1 for 96 hours at which point cell viability was quantified. The dose response was done in the absence (**A**) and presence (**B**) of exogenous FGF2. Data are normalized to the untreated control cells and are the mean ±SEM of two independent experiments resulting in the indicated *P* value.

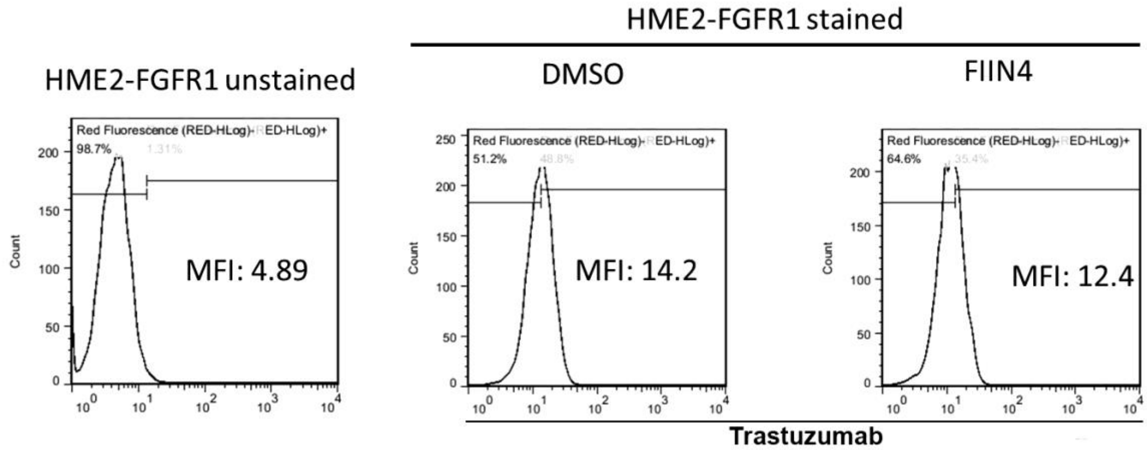

Supplementary Figure 8. Inhibition of FGFR does not increase trastuzumab binding. HME2 cells expressing FGFR1 were treated with FIIN4 and subsequently stained with trastuzumab and analyzed by flow cytometry. The resulting mean fluorescence intensities are indicated.

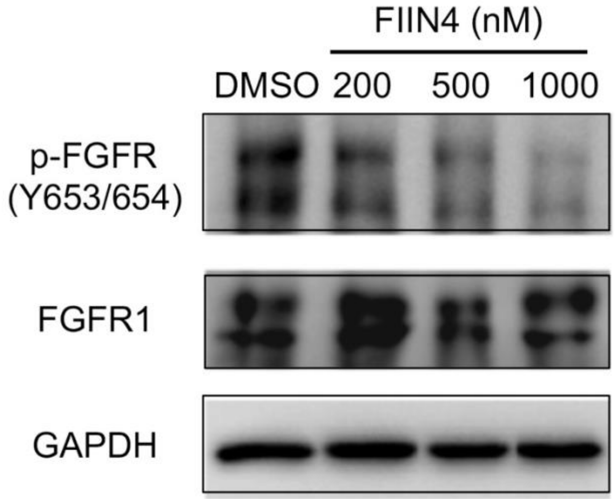

Supplementary Figure 9. Covalent inhibition of the FGFR does not degrade FGFR1. TDMR1 cells were treated with FIIN4 at the indicated concentrations for 24 hours. Total FGFR1 and phosphorylated FGFR were analyzed by immunoblot. Expression of GAPDH served as a loading control.

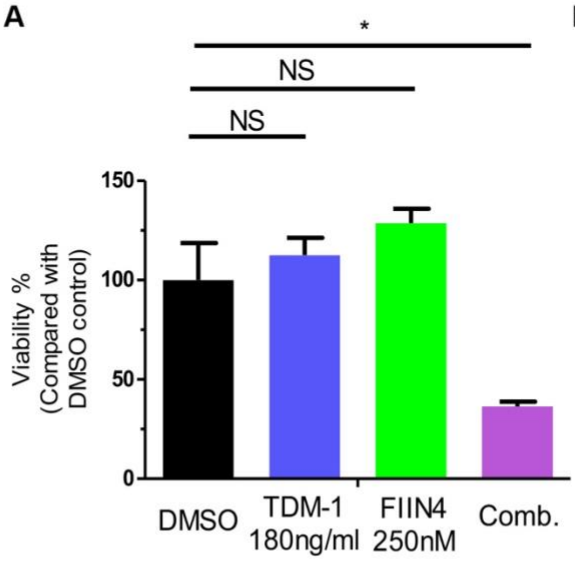

B

TDM-1	FIIN4	CI Value
180ng/ml	1250nM	0.94413
90ng/ml	625nM	0.41098
45ng/ml	312.5nM	0.34562

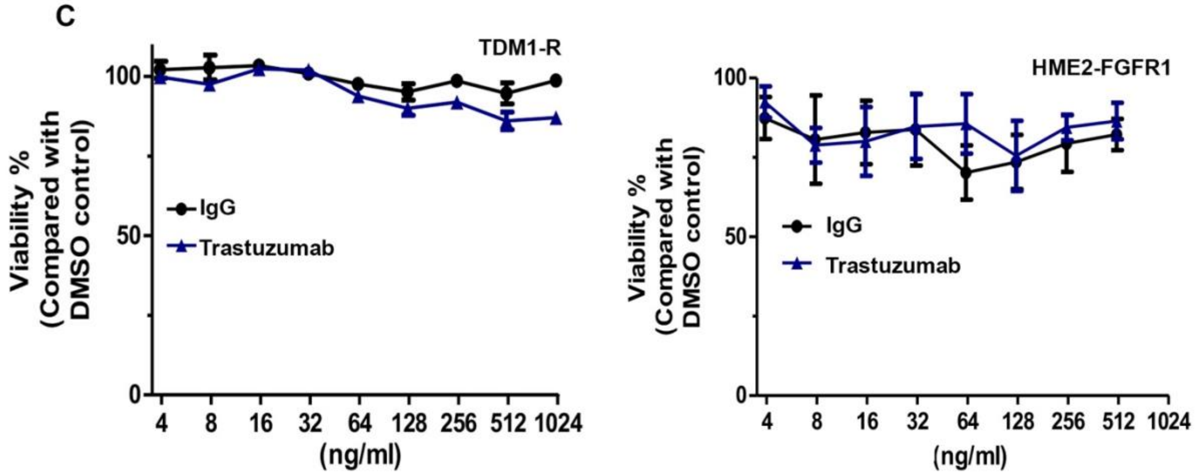

Supplementary Figure 10. Combination of TDM1 and FIIN4. **A** HME2 cells overexpressing FGFR1 were treated with various concentrations of each compound. Shown is a representative combination in which neither TDM1 nor FIIN4 alone were capable of reducing cell viability but cell viability was significantly decreased upon drug combination. **B** Additional concentrations what resulted in a synergistic combination of TDM1 and FIIN4 are listed. CI values were calculated used CompuSyn. **C** TDM1R and HME2 cells overexpressing FGFR1 were treated with the indicated concentrations of trastuzumab and analyzed for cell viability.

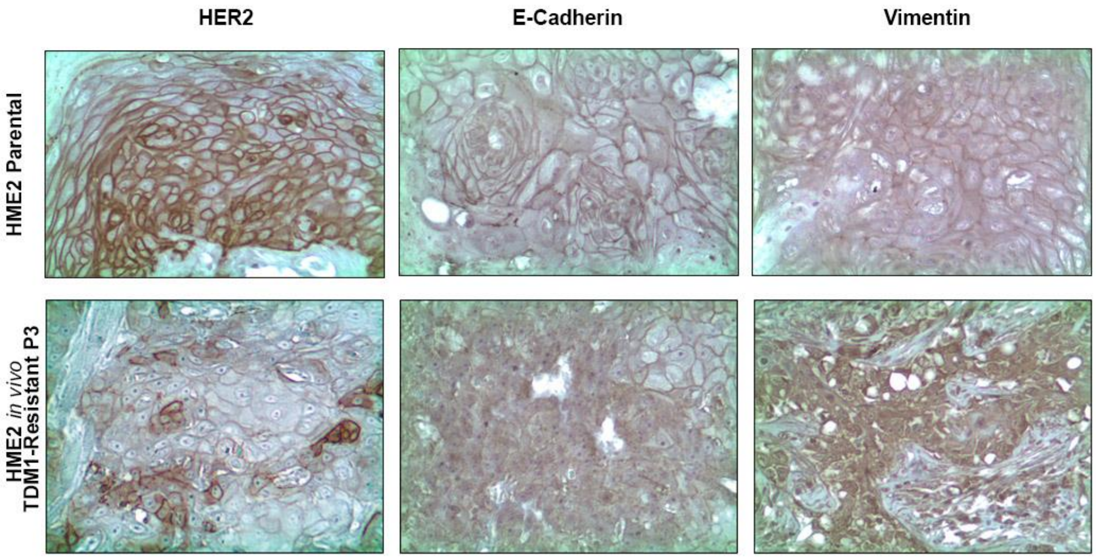

Supplementary Figure 11. IHC characterization of tumors that acquire resistance to T-DM1. As described in Figure 6, HME2 tumors were sequentially passaged *in vivo* with repeated rounds to T-DM1 treatment. The resultant T-DM1 resistant (HME2 *in vivo* TDM1-Resistant P3) tumors were analyzed by IHC for expression of HER2, E-cadherin, and Vimentin as compared to untreated HME2 parental tumors.

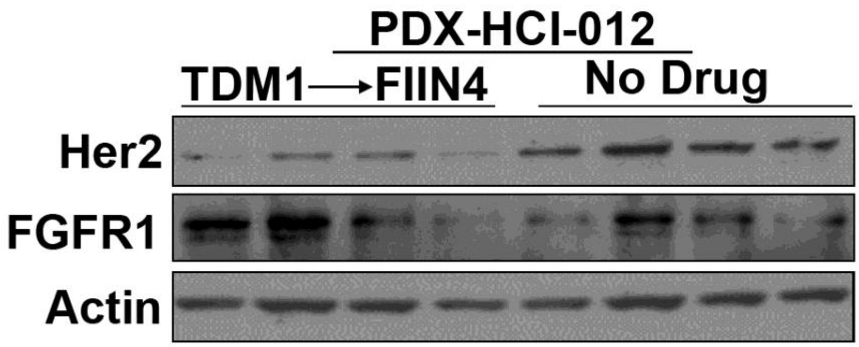

Supplementary Figure 12. Immunoblot analysis of HER2 and FGFR1 expression in HCI-012 *ex vivo* tumor tissue from four different tumor-bearing mice. As indicated, mice were either not treated (No Drug) or were treated with T-DM1, followed by FIIN4 as described in Figure 7.

Supplemental Data 1. A list of differentially phosphorylated peptides from protein tyrosine kinases (PTK) and serine threonine kinases (STK). Triplicate values were obtained from TGF-β1 pretreated cells (TGFβ) and the resulting T-DM1 resistant cells (TDM1-R). Peptides are ranked according to changes in phosphorylation levels between the two cell lines.
