# Supplementary material for: Fibroblast growth factor receptor facilitates recurrence of minimal residual disease following trastuzumab emtansine therapy
Source: NPJ Breast Cancer. 2021 Jan 21;7:5. doi: 10.1038/s41523-020-00213-5 (PMC7820437; doi:10.1038/s41523-020-00213-5)
Supplement: Supplementary file 3 — Reporting Summary Checklist [file 41523_2020_213_MOESM3_ESM.pdf]

nature research

Corresponding author(s): Mike Wendt

Last updated by author(s): Oct 23, 2020

Reporting Summary

Nature Research wishes to improve the reproducibility of the work that we publish. This form provides structure for consistency and transparency in reporting. For further information on Nature Research policies, see our Editorial Policies and the Editorial Policy Checklist.

Statistics

For all statistical analyses, confirm that the following items are present in the figure legend, table legend, main text, or Methods section.

n/a	Confirmed	
☐	☒	The exact sample size (*n*) for each experimental group/condition, given as a discrete number and unit of measurement
☐	☒	A statement on whether measurements were taken from distinct samples or whether the same sample was measured repeatedly
☐	☒	The statistical test(s) used AND whether they are one- or two-sided<br>*Only common tests should be described solely by name; describe more complex techniques in the Methods section.*
☐	☒	A description of all covariates tested
☐	☒	A description of any assumptions or corrections, such as tests of normality and adjustment for multiple comparisons
☐	☒	A full description of the statistical parameters including central tendency (e.g. means) or other basic estimates (e.g. regression coefficient) AND variation (e.g. standard deviation) or associated estimates of uncertainty (e.g. confidence intervals)
☒	☐	For null hypothesis testing, the test statistic (e.g. *F*, *t*, *r*) with confidence intervals, effect sizes, degrees of freedom and *P* value noted<br>*Give P values as exact values whenever suitable.*
☒	☐	For Bayesian analysis, information on the choice of priors and Markov chain Monte Carlo settings
☒	☐	For hierarchical and complex designs, identification of the appropriate level for tests and full reporting of outcomes
☒	☐	Estimates of effect sizes (e.g. Cohen's *d*, Pearson's *r*), indicating how they were calculated

Our web collection on statistics for biologists contains articles on many of the points above.

Software and code

Policy information about availability of computer code

Data collection	Aura imaging software for bioluminescent images, LiCor imaging software for immunoblots, Promega sofware for in vitro luminescent assays, EVOS FL software for image collection, GuavaSoft software for collection of flow cytometry data
Data analysis	PRIZM 7 for numerical data analysis, FlowJo for analysis of flow cytometry data.

For manuscripts utilizing custom algorithms or software that are central to the research but not yet described in published literature, software must be made available to editors and reviewers. We strongly encourage code deposition in a community repository (e.g. GitHub). See the Nature Research guidelines for submitting code & software for further information.

Data

Policy information about availability of data

All manuscripts must include a data availability statement. This statement should provide the following information, where applicable:

- Accession codes, unique identifiers, or web links for publicly available datasets
- A list of figures that have associated raw data
- A description of any restrictions on data availability

All data in contained within the manuscript.

Field-specific reporting

Please select the one below that is the best fit for your research. If you are not sure, read the appropriate sections before making your selection.

☒ Life sciences ☐ Behavioural & social sciences ☐ Ecological, evolutionary & environmental sciences

For a reference copy of the document with all sections, see nature.com/documents/nr-reporting-summary-flat.pdf

Life sciences study design

All studies must disclose on these points even when the disclosure is negative.

Sample size	Sample sizes were determined based on variance from previous experimental conditions.
Data exclusions	No data were excluded.
Replication	All analyses were successfully replicated.
Randomization	Samples were organized into experimental groups in a random fashion. For mouse studies, tumor bearing animals were organized into groups to ensure similar averages and variances between groups prior initiation of drug treatment.
Blinding	Blinding was not in the study to ensure proper identification, treatment and labeling of each experimental group.

Reporting for specific materials, systems and methods

We require information from authors about some types of materials, experimental systems and methods used in many studies. Here, indicate whether each material, system or method listed is relevant to your study. If you are not sure if a list item applies to your research, read the appropriate section before selecting a response.

Materials & experimental systems

n/a	Involved in the study	
☐	☒	Antibodies
☐	☐	Eukaryotic cell lines
☒	☐	Palaeontology and archaeology
☐	☒	Animals and other organisms
☒	☐	Human research participants
☒	☐	Clinical data
☒	☐	Dual use research of concern

Methods

n/a	Involved in the study	
☒	☐	ChIP-seq
☐	☒	Flow cytometry
☒	☐	MRI-based neuroimaging

Antibodies

Antibodies used	This information in found on page 7 in the manuscript in the materials and methods section.
Validation	Antibodies have been validated by molecular depletion of the intended targets.

Eukaryotic cell lines

Policy information about cell lines

Cell line source(s)	ATCC
Authentication	RADIL Cell Check
Mycoplasma contamination	IDEXX mycoplasma check
Commonly misidentified lines (See ICLAC register)	No commonly misidentified cell lines were used in this study.

Animals and other organisms

Policy information about studies involving animals; ARRIVE guidelines recommended for reporting animal research

Laboratory animals	The study included immune deficient NSG mice, female, of 4-6 weeks in age.

Wild animals	This study did not involve wild animals
Field-collected samples	The study did not involve field collected samples.
Ethics oversight	The ethics oversight for animal work was provide by the Purdue University animal use and care committee (PACUC).

Note that full information on the approval of the study protocol must also be provided in the manuscript.

Flow Cytometry

Plots

Confirm that:

☒ The axis labels state the marker and fluorochrome used (e.g. CD4-FITC).

☒ The axis scales are clearly visible. Include numbers along axes only for bottom left plot of group (a 'group' is an analysis of identical markers).

☒ All plots are contour plots with outliers or pseudocolor plots.

☒ A numerical value for number of cells or percentage (with statistics) is provided.

Methodology

Sample preparation	Cultured cells were collected and stain with trastuzumab.
Instrument	Guava EasyCyte
Software	Guava soft software was was to collect samples and these data were analyzed using flowJo.
Cell population abundance	No sorting or gating was used. All analyses shown are the entire populations.
Gating strategy	Positive and negative gates were set based on unstained controls.

☒ Tick this box to confirm that a figure exemplifying the gating strategy is provided in the Supplementary Information.
